# Mechanistic Insight Into the Regulation of Immune-Related Genes Expression in Autism Spectrum Disorder

**DOI:** 10.3389/fmolb.2021.754296

**Published:** 2021-10-21

**Authors:** Hani Sabaie, Hossein Dehghani, Shadi Shiva, Mohammad Reza Asadi, Omidvar Rezaei, Mohammad Taheri, Maryam Rezazadeh

**Affiliations:** ^1^ Molecular Medicine Research Center, Tabriz University of Medical Sciences, Tabriz, Iran; ^2^ Department of Medical Genetics, Faculty of Medicine, Tabriz University of Medical Sciences, Tabriz, Iran; ^3^ Department of Molecular Medicine, School of Medicine, Birjand University of Medical Sciences, Birjand, Iran; ^4^ Pediatric Health Research Center, Tabriz University of Medical Sciences, Tabriz, Iran; ^5^ Skull Base Research Center, Loghman Hakim Hospital, Shahid Beheshti University of Medical Sciences, Tehran, Iran; ^6^ Institute of Human Genetics, Jena University Hospital, Jena, Germany

**Keywords:** autism spectrum disorder, bioinformatics analysis, competing endogenous RNA, immune response, long non-coding RNA

## Abstract

Autism spectrum disorder (ASD) is a severe neurodevelopmental disorder featuring impairment in verbal and non-verbal interactions, defects in social interactions, stereotypic behaviors as well as restricted interests. In recent times, the incidence of ASD is growing at a rapid pace. In spite of great endeavors devoted to explaining ASD pathophysiology, its precise etiology remains unresolved. ASD pathogenesis is related to different phenomena associated with the immune system; however, the mechanisms behind these immune phenomena as well as the potential contributing genes remain unclear. In the current work, we used a bioinformatics approach to describe the role of long non-coding RNA (lncRNA)-associated competing endogenous RNAs (ceRNAs) in the peripheral blood (PB) samples to figure out the molecular regulatory procedures involved in ASD better. The Gene Expression Omnibus database was used to obtain the PB microarray dataset (GSE89594) from the subjects suffering from ASD and control subjects, containing the data related to both mRNAs and lncRNAs. The list of immune-related genes was obtained from the ImmPort database. In order to determine the immune-related differentially expressed mRNAs (DEmRNAs) and lncRNAs (DElncRNAs), the limma package of R software was used. A protein-protein interaction network was developed for the immune-related DEmRNAs. By employing the Human MicroRNA Disease Database, DIANA-LncBase, and DIANA-TarBase databases, the RNA interaction pairs were determined. We used the Pearson correlation coefficient to discover the positive correlations between DElncRNAs and DEmRNAs within the ceRNA network. Finally, the lncRNA-associated ceRNA network was created based on DElncRNA-miRNA-DEmRNA interactions and co-expression interactions. In addition, the KEGG enrichment analysis was conducted for immune-related DEmRNAs found within the constructed network. This work found four potential DElncRNA-miRNA-DEmRNA axes in ASD pathogenesis, including, *LINC00472*/*hsa-miR-221-3p*/*PTPN11*, *ANP32A-IT1*/*hsa-miR-182-5p*/*S100A2*, *LINC00472*/*hsa-miR-132-3p*/*S100A2*, and *RBM26-AS1*/*hsa-miR-182-5p*/*S100A2*. According to pathway enrichment analysis, the immune-related DEmRNAs were enriched in the “JAK-STAT signaling pathway” and “Adipocytokine signaling pathway.” An understanding of regulatory mechanisms of ASD-related immune genes would provide novel insights into the molecular mechanisms behind ASD pathogenesis.

## Introduction

Autism spectrum disorder (ASD) is a neurodevelopmental disease that manifests itself early throughout life (Parellada et al., 2014). ASD includes weak interpersonal communication, social interaction, and repetitive behaviors (Carlisi et al., 2017). Although an occurrence of 1 in 160 children has been recorded worldwide (Elsabbagh et al., 2012), there are some differences in ASD epidemiology in different parts of the world (Elsabbagh et al., 2012; Baxter et al., 2015). These differences might be attributable to various identification and screening methods, differences in community identification or diagnosis, and possible risk factors (Rice et al., 2012). ASD is defined by the Diagnostic and Statistical Manual of Mental Disorders (DSM-5) as a disorder harboring two types of symptoms: ineffective communication and social connection, and repetitive and limited behavior (Association, 2013). As a result, autistic disorder, pervasive developmental disorder not otherwise specified (PDD-NOS), Asperger’s disorder, childhood disintegrative disorder, and Rett’s disorder are now included in the definition of ASD (Harstad et al., 2015). This broad spectrum creates variations in terms of the diagnosis of ASD regarding the earlier edition of DSM (Harstad et al., 2015). Many efforts have been made to establish ASD pathogenesis; nevertheless, it remains mostly mysterious. Genetic, environmental, immunological, and neurological variables are thought to have a role in ASD development (Gładysz et al., 2018). Several immunological anomalies, such as humoral and cellular immune responses, as well as molecular abnormalities, have been documented. There is evidence of changed immune functions in both PB and cerebrospinal fluid. Multiple reports postulate an involvement of neuroinflammation in ASD, which is supported by examinations on cerebrospinal fluid and brain tissue, as well as indications of microglial activation. Immune abnormalities have been seen in a significant percentage of people with ASD (Gładysz et al., 2018).

Interacting genes with their synergistic effects cause immune-related pathological events. As a result, a combined study of non-coding RNAs and protein-encoding genes might aid in elucidating the underlying immunological processes (Li et al., 2020; Tong et al., 2020). Long non-coding RNAs (lncRNAs) have a role in a variety of biological processes, including cell differentiation, immunological responses, and development (Mercer et al., 2009). They also function as competing endogenous RNAs (ceRNAs) (Tay et al., 2014). The ceRNA theory proposes cross-talks between coding RNAs and non-coding RNAs via miRNA response elements (MREs), which are miRNA complementary sequences, establishing a large-scale regulatory network in different regions of the transcriptome. According to this hypothesis, two RNA transcripts are regulated through a process mediated by ceRNAs, which results in expressing the two RNA transcripts through indirect correlation with the expression of target miRNAs. Furthermore, there is a positive correlation in the expression of these two RNA transcripts (Salmena et al., 2011). The disruption of the equilibrium of ceRNA cross-talks has been linked to a number of disorders (Sen et al., 2014). Nevertheless, it is unknown if ceRNA plays a role in AD-related immunological responses. In this study, we used a bioinformatics approach to discover the lncRNA-associated ceRNA network in PB samples of patients with ASD in order to unravel molecular regulatory mechanisms connected to ASD-associated immunological events.

## Methods

**FIGURE 1:**
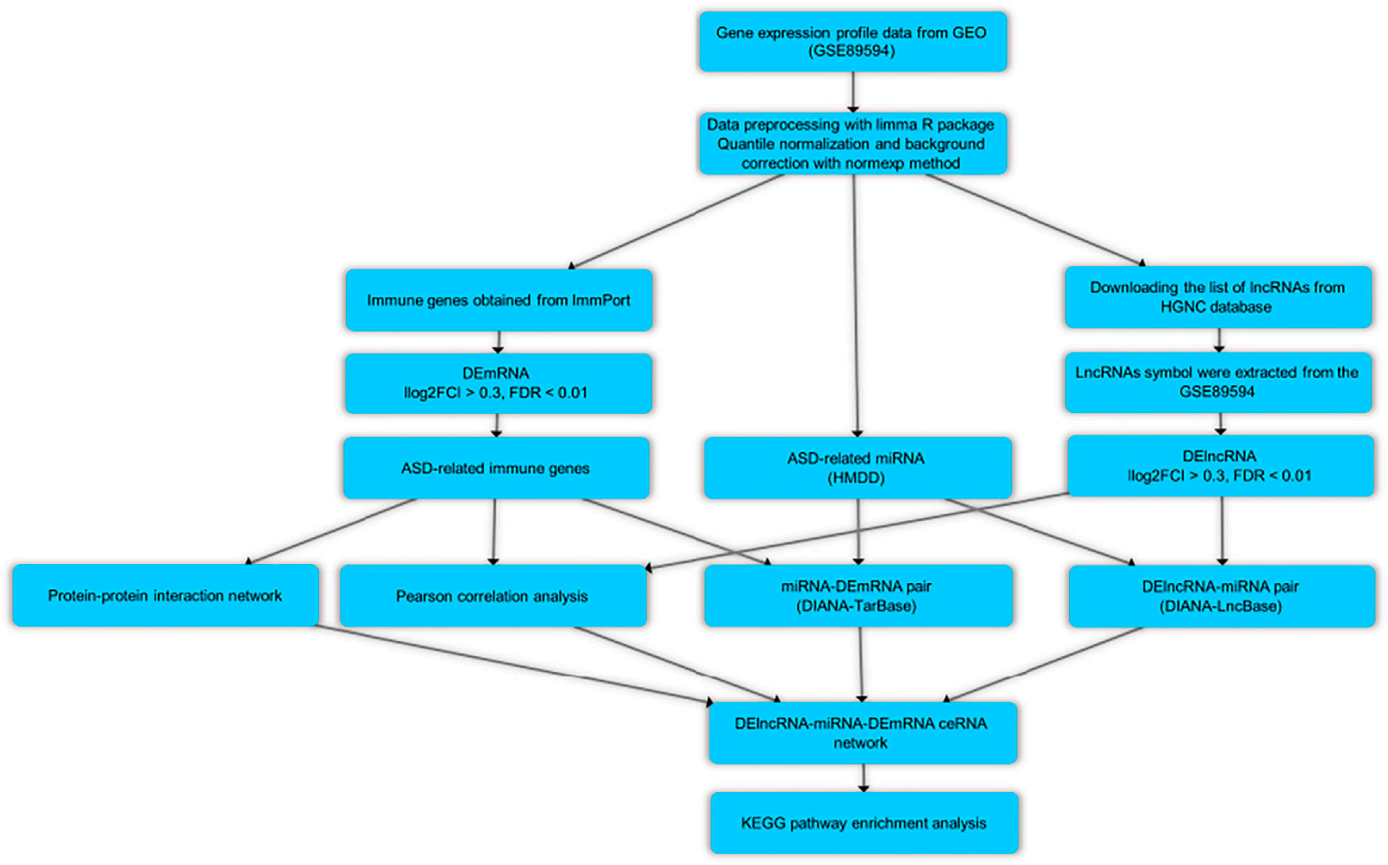
Flow chart of bioinformatics analysis.

In the current research, we used a bioinformatics method for the aim of the data mining of the microarray dataset (GSE89594) containing the PB from patients with ASD and their matched controls. We wanted to find immune-related differentially expressed mRNAs (DEmRNAs) and lncRNAs (DElncRNAs), as well as developing lncRNA-associated ceRNA regulatory network. Figure 1 depicts the steps of the bioinformatics methodology.

### Gene Expression Profile Data Collection

The above-mentioned gene expression profile was obtained from the NCBI Gene Expression Omnibus database (GEO, https://www.ncbi.nlm.nih.gov/geo/). The microarray dataset was based on the GPL16699 platform (Agilent-039494 SurePrint G3 Human GE v2 8 × 60K Microarray 039381). The GSE89594 dataset comprises 32 samples of the PB taken from individuals with ASD and 30 samples of the PB collected from healthy subjects (Kimura et al., 2019).

### Data Preprocessing and Immune-Related DEmRNAs and DElncRNAs Identification

Background correction was accomplished using the normexp method (Silver et al., 2009) with an offset of 15, and between-array normalization was accomplished using the quantile algorithm. Only spots with signal minus background flagged as “positive and significant” (field name gIsPosAndSignif) and not flagged as ControlType or IsManualFlag were used. Differentially expressed gene analysis (DEGA) was performed comparing ASD samples with normal samples by the use of linear models for microarray data (limma) R package (Ritchie et al., 2015) in Bioconductor (https://www.bioconductor.org/) (Huber et al., 2015). Age and gender were used as covariates to modify the data for confounding variables. Demographic data of ASD patients and healthy subjects are summarized in Supplementary File S1. We did a linear model on the main factor “group” (disease vs. control) using limma’s lmFit() function and included covariates in the linear model as well. The limma’s eBayes () function was then used to calculate differentially expressed data from the linear fit model. The immunologically relevant list of genes curated with functions and Gene Ontology terms was obtained from the ImmPort Portal database (https://www.immport.org/home/) (Bhattacharya et al., 2018). Further, we employed the previously described method to detect lncRNA probes (Dashti et al., 2020). The HGNC database (https://www.genenames.org/) was used to collect the entire list of lncRNA genes with HUGO Gene Nomenclature Committee (HGNC) approved symbols (Tweedie et al., 2021). Following that, we compared the list of immune-related mRNAs and lncRNAs to the gene symbols in our dataset and picked the overlapping genes. The Student t-test was employed, and the cut-offs for aberrantly expressed RNAs were established: (Parellada et al., 2014): a false discovery rate (adjusted *p*-value) < 0.01, and (Carlisi et al., 2017) |log2 fold change (log2FC)| > 0.3. The DEGs’ volcano plot and heat map were created using the Enhanced Volcano and Pheatmap R packages.

### Prediction of RNA Interaction Pairs

DIANA-LncBase v.3 was utilized to identify experimentally confirmed interactions between DElncRNAs and miRNAs (Karagkouni et al., 2020). Human microRNA Disease Database (HMDD) v3.2 was used to collect ASD-associated miRNAs (Huang et al., 2019). Moreover, we obtained experimental evidence for interactions between miRNAs collected by the use of HMDD and target mRNAs from DIANA-TarBase v.8 (Karagkouni et al., 2017). The duplicated mRNAs were used to create the DElncRNA-miRNA-DEmRNA regulatory network after the comparison of the retrieved mRNAs with the previously acquired immune-related mRNAs.

### Protein-Protein Interaction Network Analysis

PPIs were identified amongst the immune-related DEmRNAs using the Search Tool for the Retrieval of Interacting Genes/Proteins (STRING, https://string-db.org/) (Szklarczyk et al., 2019) and Molecular Interaction Search Tool (MIST) (version 5.0) (https://fgrtools.hms.harvard.edu/MIST/) (Hu et al., 2017). For PPI network development, a combined score of 0.4 (medium confidence) was selected for STRING, and None “Filter By Rank” was selected as a filter for the MIST query. The PPI network was visualized using Cytoscape software (version 3.8.0) (Shannon et al., 2003).

### Correlation Analysis Between DElncRNAs and DEmRNAs, and lncRNA-Associated ceRNA Network Construction

The Pearson correlation analysis was used to determine any positive correlations between immune-related DEmRNAs and DElncRNAs in the ceRNA regulatory network. DElncRNAs, targeted DEmRNAs, and interacted miRNAs were deleted from the ceRNA network in the opposite expression pattern between DElncRNAs and the targeted DEmRNAs. Hmisc and corrplot packages were used to compute and visualize the correlations. As inclusion criteria, Pearson correlation coefficients greater than 0.5 and *p* < 0.01 were employed. The ceRNA regulatory network were generated using Cytoscape software.

### Kyoto Encyclopedia of Genes and Genomes Pathway Enrichment Analysis

The Enrichr tool (Xie et al., 2021) was used to perform the KEGG pathway enrichment analysis for evaluating the immune-related DEmRNAs in the network. The adjusted *p*-value < 0.01 was thought to be statistically significant.

## Results

### Immune-Related DEmRNAs and DElncRNAs Identification

**FIGURE 2:**
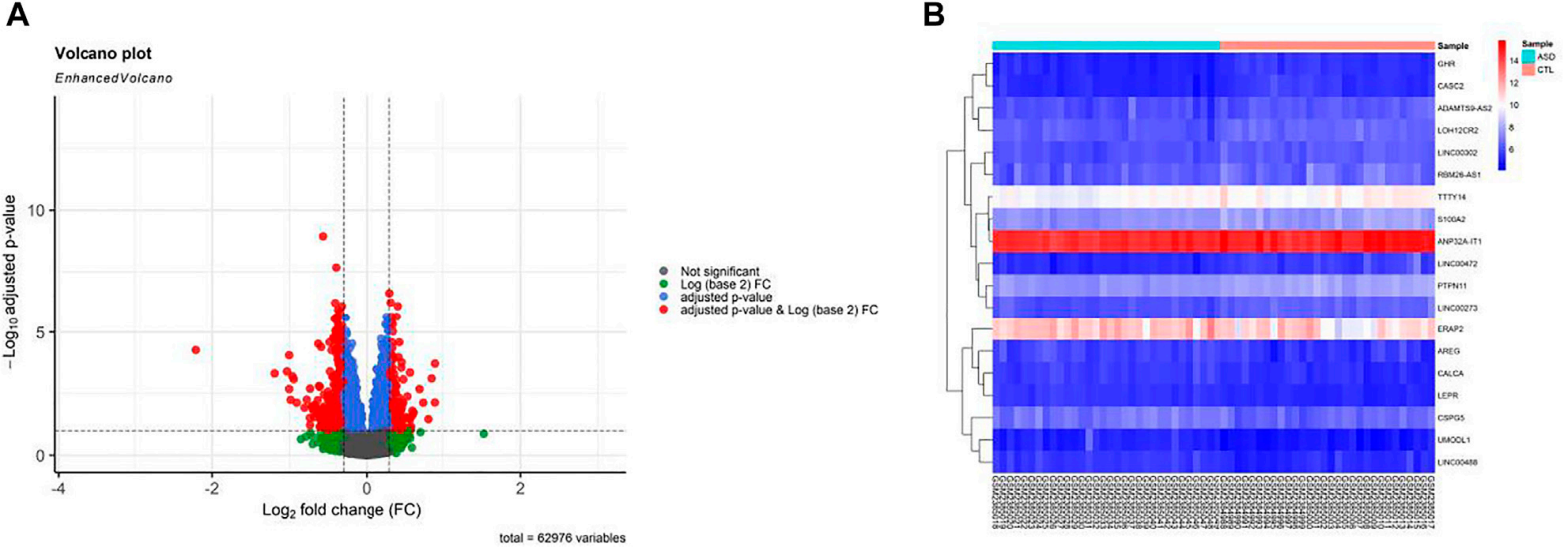
Differentially expressed genes (DEGs) between autism spectrum disorder (ASD) samples and control (CTL) samples. **(A)** Volcano plot for the DEGs. **(B)** Heatmap of immune-related DEmRNAs and DElncRNAs. High expressed genes are shown in red, while those expressed at low levels are blue. The DEGs were screened according to a |(log2FC)| > 0.3 and an adjusted *p*-value < 0.01.

Prior to conducting DEGA, background adjustments, gene filtering, normalization, and batch adjustment were performed. Box plot for gene expression-related data was presented after normalization to assess data distribution (see Supplementary File S1). In the box plot, separate arrays had identical expression level medians, demonstrating correct adjustments. According to the criteria of adjusted *p*-value < 0.01, and |(log2FC)| > 0.3, a total of 10 DElncRNAs and nine immune-related DEmRNAs were found in GSE89594 PB samples taken from ASD and healthy controls. Figure 2 depicts a volcano plot of DEGs and a heatmap of DElncRNAs and immune-related DEmRNAs. The details of DElncRNAs and immune-related DEmRNAs are summarized in Supplementary File S2.

### Prediction of RNA Interaction Pairs

We used the DIANA-LncBase v3 online platform to predict DElncRNA-miRNA interaction pairings, revealing that nine of the ten DElncRNAs may target potential miRNAs. Following that, DIANA-TarBase was used to identify relationships between miRNAs retrieved using the HMDD and potential mRNAs. We discovered eight overlapping genes after comparing the potential mRNAs to nine immune-related DEmRNAs.

### PPI Network Construction

**FIGURE 3:**
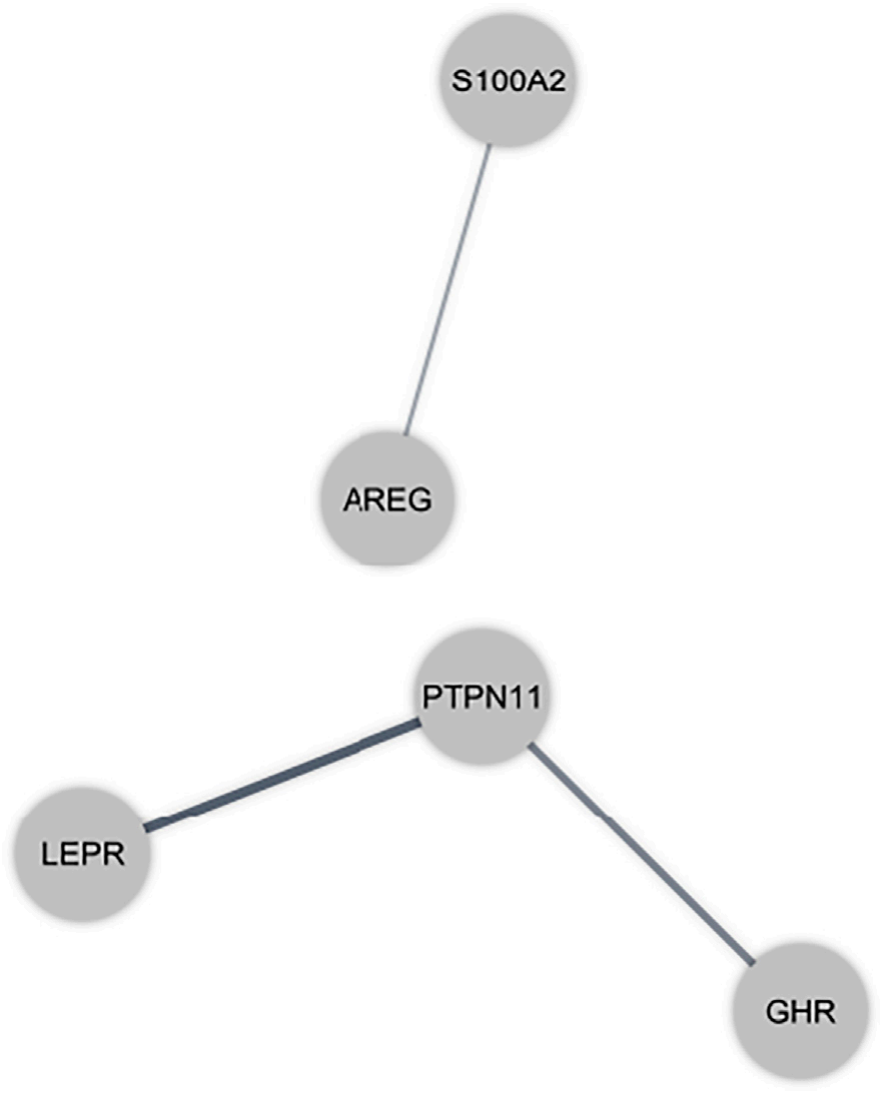
Protein-protein interaction (PPI) network of immune-related differentially expressed mRNAs (DEmRNAs) in the peripheral blood sample of autism spectrum disorder patients. Gray nodes show the interaction among DEmRNAs in the PPI network.

Protein interactions amongst immune-related DEmRNAs were identified using the online STRING and MIST tools. Figure 3 depicts the PPI network. We eliminated non-interacting genes from the PPI network to simplify it.

### Correlation Analysis Between DElncRNAs and Immune-Related DEmRNAs, and lncRNA-Associated ceRNA Network Construction

**FIGURE 4:**
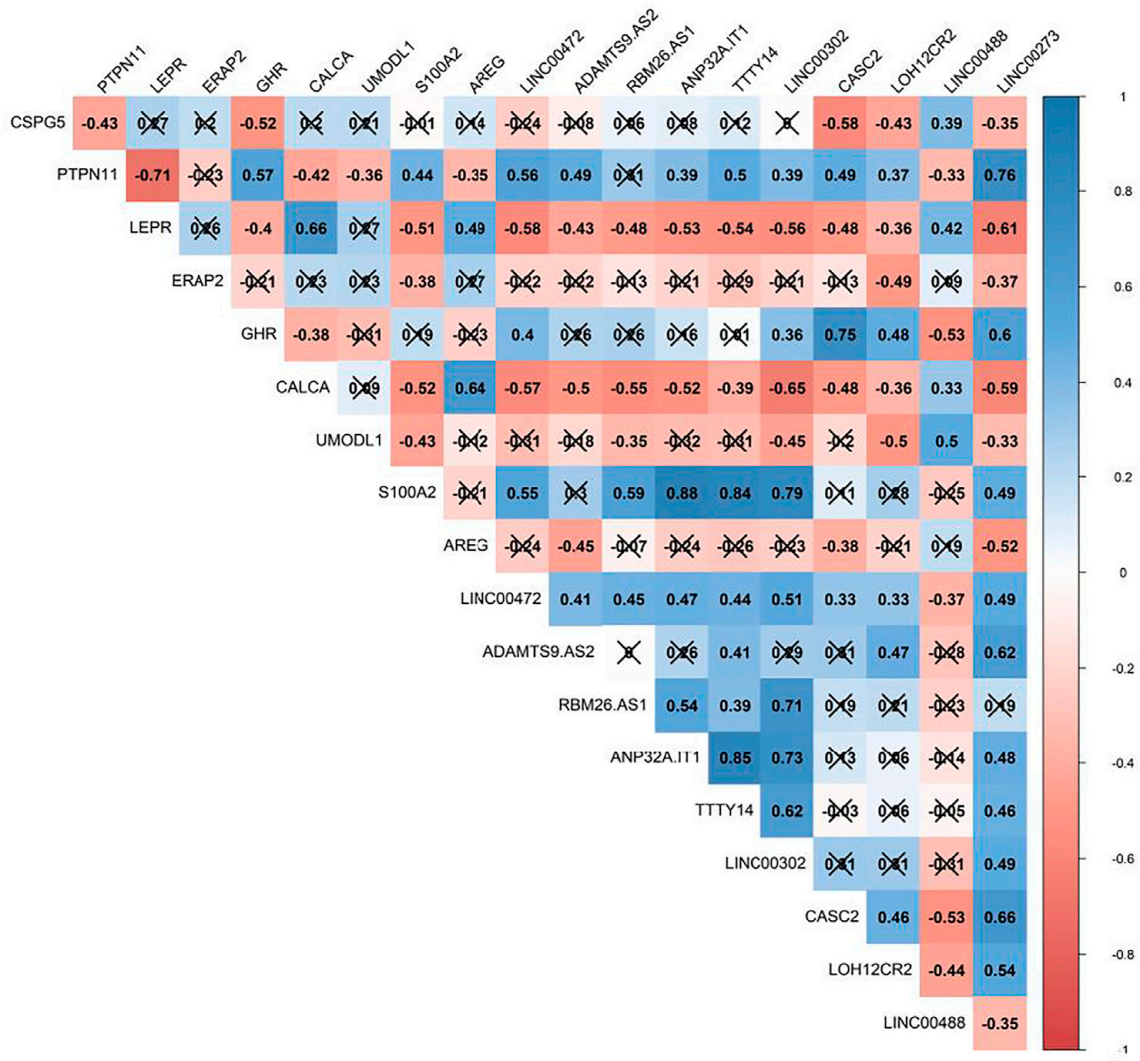
Pearson correlation analysis between immune-related differentially expressed mRNAs (DEmRNAs) and lncRNAs (DElncRNAs). The blue and red represent the positive and negative correlations, respectively. The intensity of the colors is related to correlation coefficients, and the ones with a *p*-value greater than 0.01 are considered insignificant. In this case, crosses are added.

**FIGURE 5:**
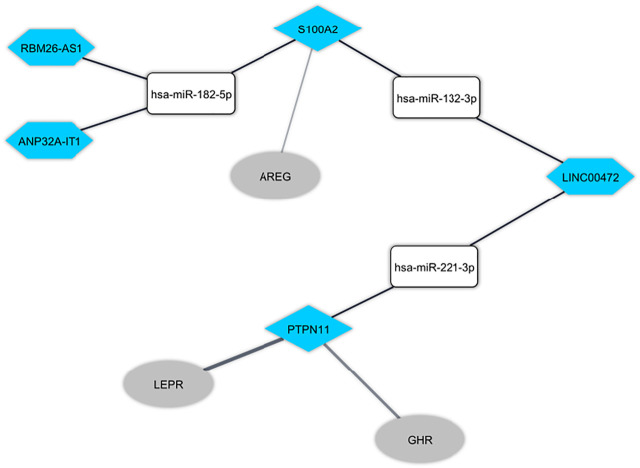
The immune-related long non-coding RNA (lncRNA)-associated competing endogenous RNA network in peripheral blood in autism spectrum disorder. The hexagon nodes and the round rectangle nodes represent the lncRNA and miRNA, respectively. The diamond nodes represent miRNAs targeted mRNAs. The ellipse nodes represent downstream connected mRNAs. The blue nodes represent the downregulation.

The Pearson correlation analysis was done between immune-related DEmRNAs and DElncRNAs to validate the ceRNA axis hypothesis, which states that lncRNA positively regulates mRNA expression by interaction with miRNA (Figure 4). We developed ceRNA regulatory network based on co-expression associations and DElncRNA-miRNA-DEmRNA interactions to explain the mechanism behind ASD development (Figure 5). Three DElncRNAs (*LINC00472*: long intergenic non-protein coding RNA 472, *ANP32A-IT1*: ANP32A intronic transcript 1, and *RBM26-AS1*: RBM26 antisense RNA 1), two immune-related DEmRNAs (*PTPN11*: protein tyrosine phosphatase non-receptor type 11 and *S100A2*: S100 calcium binding protein A2), and three miRNAs (*hsa-miR-221-3p*, *hsa-miR-182-5p*, and *hsa-miR-132-3p*) were included totally.

### KEGG Pathway Enrichment Analysis

A KEGG enrichment analysis on immune-related DEmRNAs within the network was carried out. The KEGG pathways that have been enriched were the “JAK-STAT signaling pathway” and “Adipocytokine signaling pathway.”

## Discussion

This study was concentrated on immune-related genes implicated in the development of ASD. We discovered nine immune-related DEmRNAs and created a ceRNA network in order to investigate the regulatory processes underlying mRNA, miRNA, and lncRNA interactions. Numerous studies have found that ceRNA regulatory axes plus accompanying networks are functional in a range of developmental pathways and pathological situations, including cancer, neurodegenerative diseases (Ala, 2020), and mental disorders (Lang et al., 2019; Wang et al., 2020). LncRNAs have an important function in the control of physiological and pathological processes as a significant category of RNAs in the ceRNA machinery. The expression level of lncRNAs has been discovered to be altered by a variety of mental disorders (Zuo et al., 2016). Based on the aforementioned theoretical notions, the ceRNA regulatory network associated with lncRNAs can perform critical roles in ASD etiology. So far, research studies on the ceRNA network implicated in ASD-associated immunity have been inadequate, and more study on the associated expression patterns and processes is required. In this study, we used a publicly accessible database for downloading the expression profiles related to PB samples from patients with ASD to analyze the immune-related DEmRNAs and DElncRNAs in ASD and healthy samples. Then we created a DElncRNA-miRNA-DEmRNA regulatory network. We identified four possible DElncRNA-miRNA-DEmRNA axes in the immune-related phenomena in ASD pathogenesis: *LINC00472*/*hsa-miR-221-3p*/*PTPN11*, *ANP32A-IT1*/*hsa-miR-182-5p*/*S100A2*, *LINC00472*/*hsa-miR-132-3p*/*S100A2*, and *RBM26-AS1*/*hsa-miR-182-5p*/*S100A2*.

Various studies have shown that lncRNA dysregulation may play a role in ASD etiology (Tang et al., 2017). Our research revealed three key lncRNAs (*LINC00472*, *ANP32A-IT1*, and *RBM26-AS1*) that function as ceRNAs. Several studies suggest that *LINC00472* has an essential function in the inhibition process of tumor development. In acute hepatic damage induced by sepsis and clear cell renal cell carcinoma, *LINC00472* controls immune-related genes via functioning as ceRNA for biding to various miRNAs (Li et al., 2020; Gao and Wang, 2021). *ANP32A-IT1* is the intronic transcript 1 of the *ANP32A*. *ANP32A* is widely recognized to be involved in neuritogenesis, brain development, and neuron differentiation. The regulation of *ANP32A* signaling might help control oxidative stress in the brain (Cornelis et al., 2018) and recover cognitive function (Chai et al., 2018), perhaps with therapeutic applications for neurological diseases. Even though the roles of ANP32A have been discovered, the activity of *ANP32A-IT1* is yet unknown. A recent research study showed *ANP32A-IT1* dysregulation in the PB mononuclear cells of schizophrenia patients compared to healthy subjects (Gardiner et al., 2013). *RBM26-AS1* is an antisense lncRNA, and no known function has yet been assigned to *RBM26-AS1*. Because the host transcript may be controlled by the same numbers of antisense transcripts, the adjacent *RBM26* gene might clarify the corresponding contributions of this lncRNA. *RBM26* is an RNA binding motif protein that participates in the polyadenylated RNA turnover in mammalian nuclei. The Poly(A) Tail eXosome Targeting (PAXT) connection promotes the recruiting process of the human ribonucleolytic RNA exosome to nuclear polyadenylated RNA. RBM26, as a new factor, is required for the PAXT function (Silla et al., 2020). Two previous integrated analysis studies reported that *RBM26-AS1* acts as a ceRNA in high-glucose-induced human retinal endothelial cells (Cao et al., 2020) and pancreatic cancer (Liu et al., 2020). Furthermore, in melanoma, *RBM26-AS1* dysregulation has been found in CD4 memory T cells (Zhang et al., 2021). It is noteworthy that the association between these three lncRNAs with ASD has been reported in this study for the first time; therefore, more research is required to confirm our findings.

MiRNAs attach to the untranslated area of the gene of interest, therefore regulating gene expression. MiRNAs have been found to influence biological processes and signal transduction within the cell, therefore causing ASD development (Anitha and Thanseem, 2015). The current study revealed that the key DElncRNAs could sponge three important miRNAs related to ASD (*hsa-miR-221-3p*, *hsa-miR-182-5p*, and *hsa-miR-132-3p*), leading to a regulatory effect on key DEmRNAs. According to the HMDD database, *hsa-miR-221-3p* is dysregulated in olfactory mucosal stem cells taken from ASD patients in comparison with healthy controls, and *hsa-miR-221-3p* also exhibits significant neuronal expression in areas crucial for high cognitive skills in the brain of mice (Nguyen et al., 2016). It has also been observed that *hsa-miR-182-5p* may suppress the expression of the immunity‐associated protein family 1 (*Gimap1*), shown to be reduced in T-helper cell type 17 (Th17) cells and is crucial for Th17 development (Kästle et al., 2017). Inflammation mediated by Th17 has been engaged in different diseases, including ASD (Moaaz et al., 2019). Furthermore, a previous study found that methyl CpG binding protein 2 (*MECP2*) has an analgesic function in acute and chronic pain development by modulating the *CREB*/*hsa-miR-132-3p* pathway. *MECP2* is engaged in ASD development and has been connected to neuronal differentiation, synaptic plasticity, and brain development (Zhang et al., 2015). Most of these studies confirm the current findings; however, molecular techniques (e.g., luciferase reporters, co-immunoprecipitation, and PCR) have yet to be used to validate the predicted ceRNA axes.

We found that the aforementioned lncRNAs and miRNAs regulate two key immune-related mRNAs (*PTPN11* and *S100A2*) in a ceRNA manner. *PTPN11*, also known as *SHP2*, encodes an unusual protein phosphatase functioning as an activator in different signaling pathways, such as Ras/mitogen-activated protein kinase (Ras/MAPK) signaling pathway, the downstream signaling cascades of other phosphatases are suppressed most of the time. Besides the RAS pathway, it has been demonstrated that SHP2 can also activate the PI3K-AKT pathway, whilst inhibiting the JAK-STAT pathway. In addition, the *PTPN11* gene mutations are predominantly related to RASopathies, including Noonan syndrome (NS), and cognitive deficits such as learning disabilities are common in patients with NS (Ryu et al., 2020). According to the previous works, the higher severity and prevalence of autism symptoms in RASopathies indicate that during development, the dysregulation of Ras/MAPK signaling may be implicated in ASD risk (Adviento et al., 2014). *S100A2* is a member of the EF-hand motif family S1007 (Hountis et al., 2014). The family of S100 is comprised of more than twenty members of highly-conserved acidic proteins binding to calcium with a wide range of extracellular and intracellular functions, including regulation of cell apoptosis, differentiation, proliferation, energy metabolism, inflammation, migration, protein phosphorylation, and calcium balance (Xia et al., 2018). Numerous S100 proteins are known for being overexpressed in cancers, among which S100A2 is believed to mainly act as a tumor-suppressive agent (Donato et al., 2013). Alzheimer’s disease (AD) has been shown to present a statistically significant difference in DNA methylation for *S100A2* (Siegmund et al., 2007). The changes in methylation of *S100A2* related to disease and age in AD are in agreement with finding S100A2 proteins in corpora amylacea/polyglucosan bodies accumulating in brains of aging people (Hoyaux et al., 2000). In addition, while the members of the S100A family are broadly engaged in various regulatory processes of inflammatory diseases, such as Kawasaki disease, ischemic heart inflammation, chorioamnionitis, and eye inflammation (Rohde et al., 2014; Tong et al., 2014; Wu et al., 2014; Huang et al., 2018; Golubinskaya et al., 2020), no research has been conducted on the *S100A2* gene in ASD immunity so far.

In the present study, KEGG enrichment analysis of immune-related DEmRNAs that were in the constructed network was performed as well. The enriched KEGG pathways were “JAK-STAT signaling pathway” and “Adipocytokine signaling pathway.” Cytokines are among the signaling peptides created by the stromal and immune cells, contributing to the tissue homeostasis and the immune response, such as the central nervous system (CNS) and the peripheral nervous system. In addition to serving as an immunoregulator, a number of cytokines have been considered as neuromodulators as well. Finely tuned cross-talk exists between the immune system and CNS. While the nervous system can modulate the immune response, the immune system is also capable of modulating the nervous system consistently via cytokine activity. More precisely, cytokines that activate the JAK/STAT pathway and their receptors are expressed in various brain cells. In order to maintain CNS homeostasis, both pro- and anti-inflammatory cytokines play significant roles. Different cytokines may result in activation of various sub-pathways associated with the JAK/STAT signaling. Given the significance of this pathway when the immunological responses and homeostasis are regulated in the nervous system, the alterations in JAK/STAT molecules and also the profile of numerous cytokines have implications in ASD (Baranova et al., 2021). For example, a study has referred to the increased levels of STAT5 and JAK1 within the PB mononuclear cells of the subjects with ASD (Ahmad et al., 2017), reflecting a key role in the disorder’s pathogenesis. Besides, adipocytokines (also called adipokines) are the cytokines mainly secreted via the adipose tissues with potential systemic impacts. The patients with ASD show significant changes in the adipokines levels found within the plasma. They reflect the development of systemic changes in ASD, and thus, they may be the disease hallmarks (Rodrigues et al., 2014).

There are some limitations in our study. Firstly, various parameters, such as sample preparation, the use of different methodologies, data analysis, patient characteristics, and platforms, can affect the gene expression patterns. Secondly, the small size of the sample may result in lower statistical confidence. Finally, the current findings will be validated through confirmative experimental techniques and making a comparison of the findings with modified microarray gene expression obtained from a re-analysis in future studies.

## Conclusion

In conclusion, this study is the first to identify lncRNA-associated ceRNA network based on DEmRNAs associated with immune function in ASD. Four possible DElncRNA-miRNA-DEmRNA axes, including *LINC00472*/*hsa-miR-221-3p*/*PTPN11*, *ANP32A-IT1*/*hsa-miR-182-5p*/*S100A2*, *LINC00472*/*hsa-miR-132-3p*/*S100A2*, and *RBM26-AS1*/*hsa-miR-182-5p*/*S100A2*, were identified herein. The observed results are introductory, and additional *in vitro* and *in vivo* experiments could profoundly strengthen these findings. While the potential functions of these ceRNA axes demand more in-depth investigation, this research advances the current insights into the immune processes associated with ASD and presents a new perspective into the molecular mechanisms behind ASD pathogenesis.

## Data Availability

The original contributions presented in the study are included in the article/Supplementary Material, further inquiries can be directed to the corresponding authors.
