# Supplementary material for: Mechanistic Insight Into the Regulation of Immune-Related Genes Expression in Autism Spectrum Disorder
Source: Front Mol Biosci. 2021 Oct 21;8:754296. doi: 10.3389/fmolb.2021.754296 (PMC8568055; doi:10.3389/fmolb.2021.754296)
Supplement: Supplementary file 2 [file DataSheet1.docx]

| Table S1. Demographic data of ASD patients and controls | | | |
| --- | --- | --- | --- |
| Sample | Age | Condition | Gender |
| GSM2384988 | 22y | control | female |
| GSM2384989 | 23y | control | female |
| GSM2384990 | 24y | control | female |
| GSM2384991 | 24y | control | female |
| GSM2384992 | 33y | control | male |
| GSM2384993 | 22y | control | male |
| GSM2384994 | 24y | control | female |
| GSM2384995 | 21y | control | male |
| GSM2384996 | 24y | control | male |
| GSM2384997 | 20y | control | female |
| GSM2384998 | 28y | control | female |
| GSM2384999 | 21y | control | male |
| GSM2385000 | 21y | control | female |
| GSM2385001 | 22y | control | male |
| GSM2385002 | 25y | control | male |
| GSM2385003 | 23y | control | female |
| GSM2385004 | 20y | control | female |
| GSM2385005 | 21y | control | male |
| GSM2385006 | 20y | control | female |
| GSM2385007 | 32y | control | male |
| GSM2385008 | 36y | control | female |
| GSM2385009 | 24y | control | male |
| GSM2385010 | 21y | control | male |
| GSM2385011 | 30y | control | female |
| GSM2385012 | 28y | control | male |
| GSM2385013 | 22y | control | male |
| GSM2385014 | 24y | control | female |
| GSM2385015 | 21y | control | male |
| GSM2385016 | 22y | control | male |
| GSM2385017 | 20y | control | female |
| GSM2385018 | 27y | ASD | female |
| GSM2385019 | 22y | ASD | female |
| GSM2385020 | 23y | ASD | male |
| GSM2385021 | 20y | ASD | male |
| GSM2385022 | 31y | ASD | male |
| GSM2385023 | 27y | ASD | female |
| GSM2385024 | 32y | ASD | male |
| GSM2385025 | 20y | ASD | male |
| GSM2385026 | 36y | ASD | male |
| GSM2385027 | 22y | ASD | female |
| GSM2385028 | 28y | ASD | female |
| GSM2385029 | 25y | ASD | female |
| GSM2385030 | 35y | ASD | male |
| GSM2385031 | 22y | ASD | female |
| GSM2385032 | 22y | ASD | male |
| GSM2385033 | 10y | ASD | male |
| GSM2385034 | 16y | ASD | female |
| GSM2385035 | 10y | ASD | female |
| GSM2385036 | 33y | ASD | male |
| GSM2385037 | 21y | ASD | female |
| GSM2385038 | 11y | ASD | male |
| GSM2385039 | 10y | ASD | male |
| GSM2385040 | 35y | ASD | female |
| GSM2385041 | 12y | ASD | male |
| GSM2385042 | 38y | ASD | female |
| GSM2385043 | 24y | ASD | male |
| GSM2385044 | 34y | ASD | male |
| GSM2385045 | 32y | ASD | female |
| GSM2385046 | 21y | ASD | female |
| GSM2385047 | 29y | ASD | female |
| GSM2385048 | 20y | ASD | male |
| GSM2385049 | 19y | ASD | female |
| ASD: Autism spectrum disorder | | | |


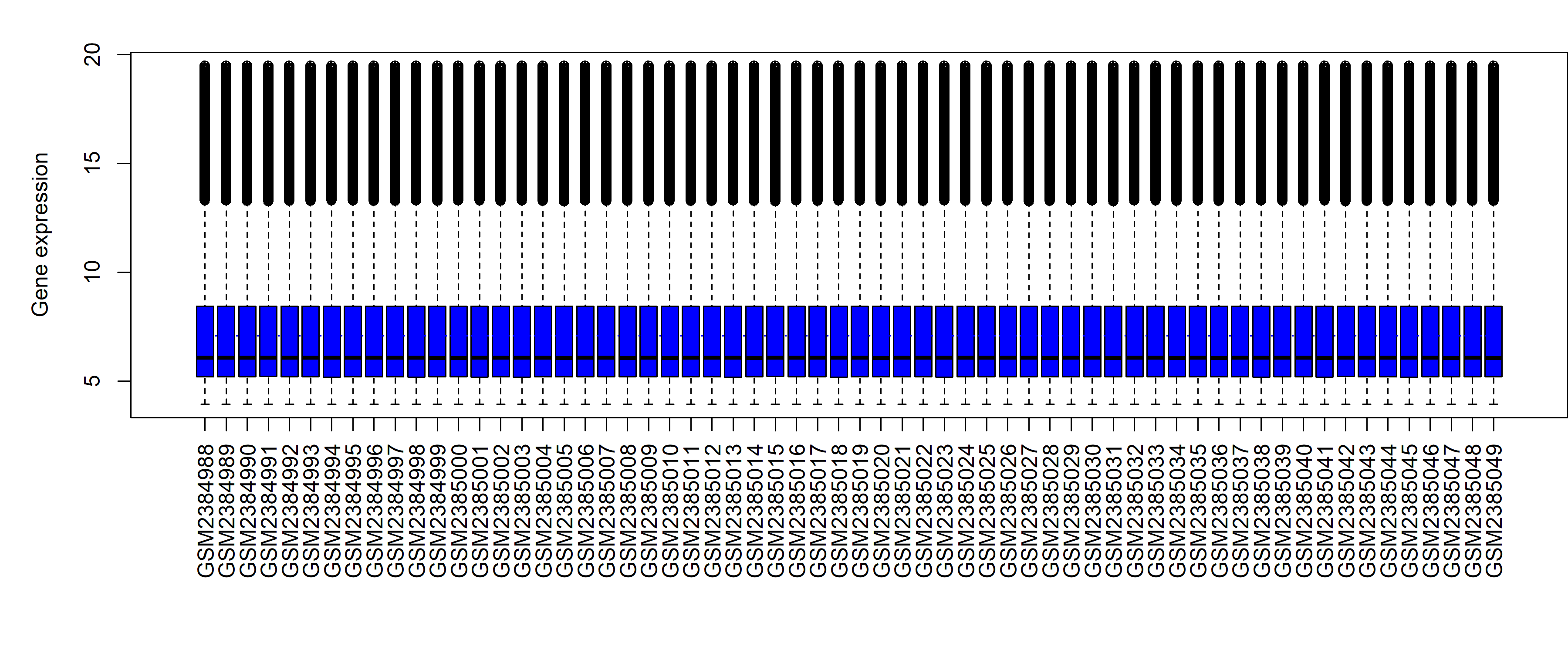
**Figure S1.** Boxplot for GSE89594 dataset. The horizontal axis represents the names of samples, and the vertical axis represents the gene expression.
